# Enhancement of Antimycotic Activity of Amphotericin B by Targeting the Oxidative Stress Response of *Candida* and *Cryptococcus* with Natural Dihydroxybenzaldehydes

**DOI:** 10.3389/fmicb.2012.00261

**Published:** 2012-07-19

**Authors:** Jong H. Kim, Natália C. G. Faria, M. De L. Martins, Kathleen L. Chan, Bruce C. Campbell

**Affiliations:** ^1^Plant Mycotoxin Research Unit, Western Regional Research Center, Agricultural Research Service, United States Department of AgricultureAlbany, CA, USA; ^2^Instituto de Higiene e Medicina Tropical/CREM, Universidade Nova de LisboaLisboa, Portugal

**Keywords:** amphotericin B, dihydroxybenzaldehydes, chemosensitization, *Candida*, *Cryptococcus*, antioxidant system, superoxide dismutase

## Abstract

In addition to the fungal cellular membrane, the cellular antioxidant system can also be a viable target in the antifungal action of amphotericin B (AMB). Co-application of certain redox-potent natural compounds with AMB actually increases efficacy of the drug through chemosensitization. Some redox-potent chemosensitizers and AMB perturb common cellular targets, resulting in synergistic inhibition of fungal growth. Chemosensitizing activities of four redox-potent benzaldehydes were tested against clinical and reference strains of *Candida albicans*, *C. krusei*, *C. tropicalis*, and *Cryptococcus neoformans* in combination with AMB, based on assays outlined by the European Committee on Antimicrobial Susceptibility Testing. Two dihydroxybenzaldehydes (DHBAs), i.e., 2,3-DHBA and 2,5-DHBA, significantly enhanced activity of AMB against most strains, as measured by lower minimum inhibitory concentrations and/or minimum fungicidal concentrations (MFCs). A non-hydroxylated benzaldehyde, *trans*-cinnamaldehyde, showed chemosensitizing activity through lower MFCs, only. Contrastingly, a methoxylated benzaldehyde (3,5-dimethoxybenzaldehyde) had no chemosensitizing activity, as all strains were hypertolerant to this compound. Bioassays using deletion mutants of the model yeast, *Saccharomyces cerevisiae*, indicated DHBAs exerted their chemosensitizing activity by targeting mitochondrial superoxide dismutase. This targeting, in turn, disrupted the ability of the yeast strains to respond to AMB-induced oxidative stress. These *in vitro* results indicate that certain DHBAs are potent chemosensitizing agents to AMB through co-disruption of the oxidative stress response capacity of yeasts. Such redox-potent compounds show promise for enhancing AMB-based antifungal therapy for candidiasis and cryptococcosis.

## Introduction

There has been a persistent effort to improve efficacy of conventional antimycotic drugs, especially for treatment of human candidiasis and cryptococcosis. Currently, liposomal amphotericin B (LAMB), AMB lipid complex, etc., are preferred for clinical therapy of these mycoses, in that conventional AMB (e.g., AMB deoxycholate) is hepatotoxic/nephrotoxic (Patel et al., 2011). The lipid-based AMBs are generally recommended for patients who are intolerant to conventional AMB, which is still administered for treatment of mycoses, such as pediatric fungal infections (Allen, 2010 and references therein). However, high doses of LAMBs cause nephrosis and other tissue-damage in murine models of invasive pulmonary aspergillosis (Clemons et al., 2011). Thus, an antifungal therapeutic strategy to reduce side effects of AMB is warranted.

Amphotericin B binds to ergosterol in the fungal plasma membrane, undermining cell membrane integrity and causing ion leakage. However, formation of channels in the fungal membrane is not the sole mode of action of AMB (Palacios et al., 2007). There is ample literature showing AMB induces oxidative damage to both ascomycete and zygomycete fungal cells (Sokol-Anderson et al., 1986; Graybill et al., 1997, and references therein; Okamoto et al., 2004; An et al., 2009; González-Párraga et al., 2011). For example, *Aspergillus terreus*, a causative agent of human invasive aspergillosis, is intrinsically resistant to AMB, compared to other aspergilli. This resistance was thought to result from lower membrane ergosterol, thus offering fewer target sites for AMB (Walsh et al., 2003). However, this resistance was later found to result from higher catalase activity, an enzyme that protects against oxidative stress. This latter finding indicated there is an alternate or additional mode of action of AMB by causing oxidative damage (Blum et al., 2008). This was further confirmed by the finding that superoxide radical-mediated oxidative damage was caused by AMB activity (Okamoto et al., 2004).

Disrupting fungal redox homeostasis and/or the antioxidant system should augment antimycotic activity of AMB. Moreover, the antioxidant system plays an important role in pathogen virulence and defense against host cellular oxidative burst during infection (Washburn et al., 1987; Hamilton and Holdom, 1999; de Dios et al., 2010). Such disruption of the fungal redox homeostasis/antioxidant system could employ redox-potent natural products or their analogs (Jacob, 2006). The natural phenolic 2,3-dihydroxybenzaldehyde (2,3-DHBA) augments antifungal activity of a number of fungicidal agents by interfering with the fungal oxidative stress response system (Kim et al., 2008, 2011). In view that both 2,3-DHBA and AMB stress the fungal antioxidant system, their co-application should result in elevated antifungal activity.

The aim of this study was to test the concept of using benzaldehydes, such as 2,3-DHBA and some of its structural derivatives, as chemosensitizing agents to AMB. As a proof-of-concept, we used clinical strains and species of *Candida* and *Cryptococcus neoformans* for this test. Specifically, we compared the chemosensitizing activity between two hydroxylated DHBAs (2,3- or 2,5-DHBA) and two non-hydroxylated benzaldehydes [non-DHBAs; *trans*-cinnamaldehyde or 3,5-dimethoxybenzaldehyde (3,5-DMBA)]. We reasoned that use of chemosensitizing agents from natural sources could enhance the activity of AMB, while lowering toxic side effects of this drug to human cells.

## Materials and Methods

### Fungal strains and culture conditions

*Candida albicans* 90028 and *C. krusei* 6258 were procured from American Type Culture Collection (Manassas, VA, USA). *C. albicans* CAN276, *C. krusei* CAN75, *C. tropicalis* CAN286 and *C. neoformans* CN24 were procured from *Instituto de Higiene e Medicina Tropical/CREM, Universidade nova de Lisboa*, Portugal. *Saccharomyces cerevisiae* wild type (WT) BY4741 (*Mat* a *his3*Δ*1* *leu2*Δ*0* *met15*Δ*0* *ura3*Δ*0*) and selected single gene deletion mutants (see text) were procured from Open Biosystems (Huntsville, AL, USA). Yeast strains were cultured on Synthetic Glucose (SG; Yeast nitrogen base without amino acids 0.67%, glucose 2% with appropriate supplements: uracil 0.02 mg mL^−1^, amino acids 0.03 mg mL^−1^) or yeast peptone dextrose (YPD; Bacto yeast extract 1%, Bacto peptone 2%, glucose 2%) agar at 30°C for *S. cerevisiae* or 35°C for yeast pathogens (*Candida*, *Cryptococcus*), respectively.

### Antifungal drugs and compounds

Amphotericin B, diamide, 2,3- or 2,5-DHBA, *trans*-cinnamaldehyde, and 3,5-DMBA were procured from Sigma Co. (St. Louis, MO, USA). Each compound was dissolved in dimethyl sulfoxide (DMSO; absolute DMSO amount: <2% in medium) before incorporation into the culture medium. In all tests, control plates (i.e., “No treatment”) contained DMSO at levels equivalent to that of cohorts receiving antifungal agents, within the same set of experiments.

### Susceptibility testing: Plate (agar) bioassay

Petri plate-based yeast dilution bioassays were performed on the WT and antioxidant mutants of *S. cerevisiae* to assess the effects of AMB (0.0, 0.5, 1.0, 1.5, and 2.0 μg mL^−1^) on the fungal antioxidant system. These assays were performed in duplicate on SG agar following previously described protocols (Kim et al., 2008). Similar dilution bioassays were performed on *Candida* and *Cryptococcus* to assess their differential sensitivity to AMB (0.0, 0.5, 1.0 μg mL^−1^) or diamide (0.0, 0.2, 0.4, 0.6, 0.8 mM). Cell growth was observed for 3–5 days.

### Susceptibility testing: Microtiter (liquid) bioassay

To determine changes in antifungal minimum inhibitory concentrations (MICs), i.e., differences/changes in MICs of each compound (AMB, benzaldehydes) alone as compared to when they were combined, triplicate assays were performed using broth microdilution protocols according to methods outlined by the European Committee on Antimicrobial Susceptibility Testing (EUCAST; Arendrup et al., 2012; definitive document EDef 7.2.). MIC was defined as the concentration at which no fungal growth was visible. These assays were performed using a range of concentrations of test compounds, as follows: AMB – 0.0, 1.0, 2.0, 4.0, 8.0, 16.0, 32.0 μg mL^−1^; 2,3-DHBA, 2,5-DHBA, *trans*-cinnamaldehyde, 3,5-DMBA – 0.0, 0.00625, 0.0125, 0.025, 0.05, 0.1, 0.2, 0.4, 0.8, 1.6, 3.2, 6.4 mM.

To measure changes in minimum fungicidal concentrations (MFCs), i.e., differences/changes of MFCs of each compound (AMB, benzaldehydes) alone compared to when they were combined, the entire volume of each microtiter well (200 μL), after determination of MICs, was spread onto individual YPD plates and cultured an additional 48 h (72 h for *C. neoformans*). The lowest concentration of agent showing ≥99.9% fungal death was defined as the MFC, except where noted (see tables). Student’s *t*-test for paired data was used to determine significant differences between means of MICs or MFCs of each compound when combined (i.e., chemosensitization) vs alone (i.e., no chemosensitization) for six yeast pathogens (calculation was based on Kirkman, 1996). Compound interactions [for both fractional inhibitory concentration indices (FICI) and fractional fungicidal concentration indices (FFCI)] were calculated based on: FICI or FFCI = (MIC or MFC of compound A in combination with compound B/MIC or MFC of compound A, alone) + (MIC or MFC of compound B in combination with compound A/MIC or MFC of compound B, alone). FICI or FFCI was defined as: “synergistic” (FICI or FFCI ≤0.5) or “indifferent” (FICI or FFCI >0.5–4; Odds, 2003).

## Results

**Figure 1:**
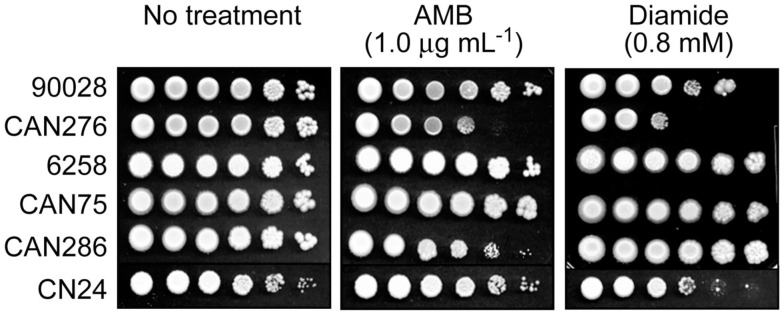
**Dilution bioassays showing phenotypic responses of yeast pathogens to amphotericin B (AMB) or diamide. 1 × 10^6^ cells were serially diluted 10-fold in SG liquid medium, and were inoculated onto agar plates**. Data are representative results shown from 1 μg mL^−1^ (AMB) and 0.8 mM (diamide), respectively.

We tested the hypothesis that benzaldehydes could act as chemosensitizing agents to AMB against clinical strains and species of *Candida* and *C. neoformans*. First, Petri plate-based yeast dilution bioassays were used to evaluate any relationship between AMB-sensitivity and lower antioxidant capacity. Duplicate assays were performed on SG agar containing AMB (0.0, 0.5, and 1.0 μg mL^−1^) according to described protocols (Kim et al., 2008). In this test, *C. albicans* CAN276 was the most sensitive of all strains when exposed up to 1.0 μg mL^−1^ AMB (Figure 1). Next, we examined the effect of diamide (0.0, 0.2, 0.4, 0.6, and 0.8 mM) on these strains. Diamide causes stoichiometric oxidative stress by depleting cellular thiols, such as glutathione. CAN276 was also the most sensitive of *Candida* species or strains to diamide (up to 0.8 mM; Figure 1). *C. krusei* 6258, *C. krusei* CAN75, and *C. tropicalis* CAN286 grew similar to control (no diamide) cohorts (i.e., no antifungal activity against these strains at the given concentration). *C. albicans* 90028 and *C. neoformans* CN24 showed slight sensitivity to diamide, >100-fold less than CAN276 (Figure 1). The high sensitivity of CAN276 to both AMB and diamide indicated a diminished oxidative stress response system increases sensitivity to AMB.

**Figure 2:**
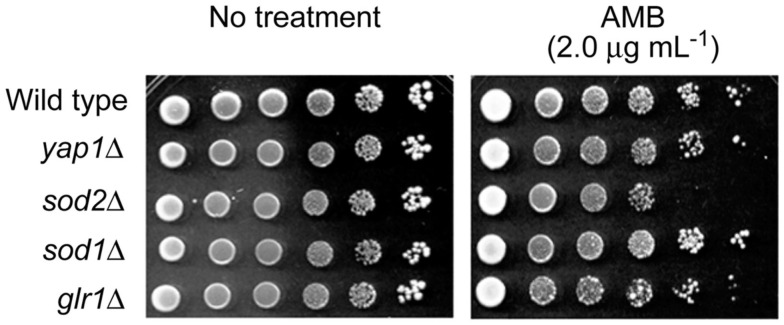
**Dilution bioassay showing phenotypic responses of *S. cerevisiae* strains to amphotericin B (AMB)**. *S. cerevisiae sod2*Δ [mitochondrial superoxide dismutase (Mn-SOD) gene deletion] mutant exhibited increased sensitivity to AMB over that of other deletion mutants (*yap1*Δ, *sod1*Δ, and *glr1*Δ) involving antioxidation responses. Data are representative results shown from 2 μg mL^−1^ (AMB). 1 × 10^6^ cells were serially diluted 10-fold in SG liquid medium, and were inoculated onto agar plates.

Identification of target(s) of AMB within the yeast antioxidant system was attempted using deletion mutants of the model fungus, *S. cerevisiae*. Petri plate-based cell-dilution bioassays on SG agar with AMB (0.0, 0.5, 1.0, 1.5, and 2.0 μg mL^−1^; in duplicate) included the WT and four antioxidant mutant strains, as follows: (1) *yap1*Δ [Yap1p is the transcription factor regulating expression of four downstream genes within the oxidative stress response pathway, i.e., *GLR1* (glutathione reductase), *YCF1* (a glutathione *S*-conjugate pump), *TRX2* (thioredoxin), and *GSH1* (γ-glutamylcysteine synthetase; Fernandes et al., 1997; Lee et al., 1999)]; (2) *sod1*Δ (cytosolic superoxide dismutase); (3) *sod2*Δ (mitochondrial superoxide dismutase, Mn-SOD); and (4) *glr1*Δ (glutathione reductase; see *Saccharomyces* Genome Database; www.yeastgenome.org, accessed May 22, 2012). These representative mutants were selected because: (1) they play key roles in maintaining cellular redox homeostasis in both enzymatic (e.g., superoxide radical-scavenging) and non-enzymatic (e.g., glutathione homeostasis) aspects; (2) among 45 *S. cerevisiae* antioxidant/stress response system mutants examined, tolerance to redox-potent benzo analogs relied upon Mn-SOD (*SOD2*) or glutathione reductase (*GLR1*; Kim et al., 2008); and (3) oxidative damage from AMB in *C. albicans* is induced by superoxide (Okamoto et al., 2004). Of the four deletion mutants, only *sod2*Δ was hypersensitive to AMB (up to 2.0 μg mL^−1^; Figure 2). These results showed Mn-SOD plays a relatively greater role in fungal tolerance to AMB-induced toxicity than the other genes represented, similar to that found for treatment by redox-potent 2,3-DHBA (Kim et al., 2008).

The capacity of benzaldehyde analogs (DHBAs and non-DHBAs) to influence antifungal activity of AMB was examined using triplicate checkerboard microdilution bioassays according to the EUCAST (see Materials and Methods). The four benzaldehydes tested were 2,3- and 2,5-DHBAs, *trans*-cinnamaldehyde, and 3,5-DMBA. All four of these compounds targeted Mn-SOD in *S. cerevisiae* (Kim et al., 2008, 2011). In prior studies, 2,3-DHBA and cinnamaldehyde exhibited the highest antifungal activity against *S. cerevisiae* or filamentous fungi, respectively, when treated alone: *S. cerevisiae*- 2,3-DHBA (MIC 0.08 mM) >2,5-DHBA (MIC 1.8 mM) or filamentous fungi- cinnamaldehyde (MIC 0.58 mM) >3,5-DMBA (MIC 1.17 mM; Kim et al., 2008, 2011). In the present study, the DHBAs had the most potent chemosensitizing activity to AMB (see Tables 1 and 2).

**Table 1:** Chemosensitization of AMB by 2,3-DHBA*.

Strains	Compounds	MIC alone	MIC combined	FICI	MFC alone	MFC combined	FFCI
*C. albicans*	2,3-DHBA	0.4	0.2	1.0	6.4	0.8	**0.4**
ATCC 90028	AMB	2	1		4	1	
*C. albicans*	2,3-DHBA	0.4	0.0125	**0.5**	3.2	0.4	0.6
CAN276	AMB	2	1		2	1	
*C. krusei*	2,3-DHBA	0.8	0.2	0.8	6.4	3.2	1.0
ATCC 6258	AMB	2	1		2	1	
*C. krusei*	2,3-DHBA	0.8	0.4	1.0	6.4	3.2	0.8
CAN75	AMB	2	1		4	1	
*C. tropicalis*	2,3-DHBA	0.8	0.2	0.8	3.2	1.6	0.8
CAN286	AMB	2	1		4	1	
*C. neoformans*	2,3-DHBA	0.8	0.4	1.0	3.2	0.1	**0.5**
CN24	AMB	4	2		4	2	
*t*-Test	2,3-DHBA		*P* < 0.005			*P* < 0.01	
	AMB		*P* < 0.05			*P* < 0.005

*MFCs are concentrations where ≥99.9% fungal death was achieved. Synergistic interactions are in **bold** (see Materials and Methods for calculations)*.

*2,3-DHBA, 2,3-dihydroxybenzaldehyde (mM); AMB, amphotericin B (μg mL^−1^); MIC, minimum inhibitory concentration; MFC, minimum fungicidal concentration; FICI, fractional inhibitory concentration indices; FFCI, fractional fungicidal concentration indices.

**Table 2:** Chemosensitization of AMB by 2,5-DHBA*.

Strains	Compounds	MIC alone	MIC combined	FICI	MFC alone	MFC combined	FFCI
*C. albicans*	2,5-DHBA	1.6	0.8	1.0	6.4	3.2	0.8
ATCC 90028	AMB	2	1		4	1	
*C. albicans*	2,5-DHBA	1.6	0.8	1.0	6.4	3.2	1.0
CAN276	AMB	2	1		2	1	
*C. krusei*	2,5-DHBA	3.2	3.2	2.0	>6.4^†^	6.4	0.8
ATCC 6258	AMB	2	2		4	1	
*C. krusei*	2,5-DHBA	3.2	0.0125	**0.5**	>6.4^†^	6.4	1.0
CAN75	AMB	4	2		4	2 (99.7% killing)	
*C. tropicalis*	2,5-DHBA	3.2	1.6	1.0	>6.4^†^	3.2	0.8
CAN286	AMB	2	1		4	2	
*C. neoformans*	2,5-DHBA	3.2	1.6	1.0	6.4	3.2	1.0
CN24	AMB	2	1		2	1	
*t*-Test	2,5-DHBA		*P* < 0.05			*P* < 0.01	
	AMB		*P* < 0.05			*P* < 0.005

*MFCs are concentrations where ≥99.9% fungal death was achieved, except where noted in the table. Synergistic interactions are in **bold** (see Materials and Methods for calculations)*.

*2,5-DHBA, 2,5-dihydroxybenzaldehyde (mM); AMB, amphotericin B (μg mL^−1^); MIC, minimum inhibitory concentration; MFC, minimum fungicidal concentration; FICI, fractional inhibitory concentration indices; FFCI, fractional fungicidal concentration indices.

*^†^Assays were conducted up to the highest concentration of 6.4 mM. For calculation purposes, 12.8 mM (doubling of 6.4 mM) was used*.

**Figure 3:**
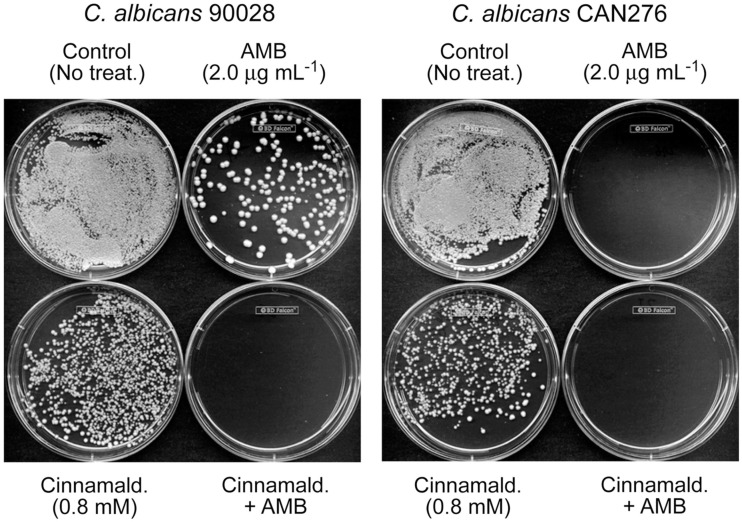
**Exemplary plate bioassay to determine minimum fungicidal concentration (MFC) in *C. albicans* 90028 (a reference strain) and CAN276 (a clinical isolate)**. In *C. albicans* 90028, co-application of AMB (2.0 μg mL^−1^) and cinnamaldehyde (0.8 mM) completely inhibited colony survival, while survived colonies appeared under the independent treatment of AMB or cinnamaldehyde. Similar assay was performed on *C. albicans* CAN276, where no colonies appeared on 2.0 μg mL^−1^ of AMB, confirming the higher sensitivity of *C. albicans* CAN276 to AMB than *C*. *albicans* 90028.

As an example of DHBA-AMB interactions, the MIC for AMB (MIC_AMB_), alone, for *C. albicans* 90028 was 2 μg mL^−1^ (Tables 1 and 2). However, the MIC_AMB_ was lowered to <1 μg mL^−1^ with either of the DHBAs. MICs of the DHBAs were concomitantly lowered in these co-applications, as well. MFCs were similarly affected, where the MFC of AMB alone (4 μg mL^−1^) was reduced to <1 μg mL^−1^ by co-treatment with DHBAs. The relatively higher sensitivity of CAN276 than *C*. *albicans* 90028 to AMB (see Figure 1) was also reflected in MFC values; MFC_AMB_ CAN276 = 2 μg mL^−1^, MFC_AMB_ *C*. *albicans* 90028 = 4 μg mL^−1^ (Tables 1 and 2; See also Figure 3). The range of MICs of 2,3-DHBA (0.4–0.8 mM) was lower than that of 2,5-DHBA (1.6–3.2 mM) in all yeasts tested (Tables 1 and 2). Thus, the higher to lower antifungal activity of 2,3-DHBA >2,5-DHBA in these yeast pathogens reflected that of *S*. *cerevisiae* (Kim et al., 2008; see also above).

The non-DHBAs tested were not potent chemosensitizing agents for AMB against the yeasts, as compared with the DHBAs. Interactions of cinnamaldehyde co-applied with AMB, in *C*. *albicans* 90028, CAN276, *C*. *krusei* 6258, and *C*. *neoformans* CN24, were “indifferent,” although this co-application showed certain level of enhanced antifungal activity for MFCs (Table 3). Moreover, 3,5-DMBA did not show any antifungal activity in any of the yeast strains, even at the highest concentration tested (6.4 mM), nor any chemosensitization when co-applied with AMB (data not shown). Contrastingly, 3,5-DMBA had potent antifungal activity (average MIC: 1.17 mM) against filamentous fungal pathogens (i.e., species and strains of *Aspergillus*, *Penicillium*; Kim et al., 2011). Perhaps yeast pathogens possess an intrinsic capacity to detoxify 3,5-DMBA.

**Table 3:** Chemosensitization of AMB by cinnamaldehyde*.

Strains	Compounds	MIC alone	MIC combined	FICI	MFC alone	MFC combined	FFCI
*C. albicans*	Cinn	0.8	0.8	2.0	1.6	0.8	0.8
ATCC 90028	AMB	2	2		4	1	
*C. albicans*	Cinn	0.8	0.8	2.0	1.6	0.8	1.0
CAN276	AMB	2	2		2	1	
*C. krusei*	Cinn	0.8	0.8	2.0	1.6	0.8	1.0
ATCC 6258	AMB	4	4		4 (99.8% killing)	2	
*C. krusei*	Cinn	0.8	0.8	2.0	0.8	0.8	2.0
CAN75	AMB	4	4		4	4	
*C. tropicalis*	Cinn	1.6	1.6	2.0	1.6	1.6	2.0
CAN286	AMB	2	2		4	4	
*C. neoformans*	Cinn	0.8	0.8	2.0	0.8	0.4	1.0
CN24	AMB	4	4		4	2 (99.8% killing)	
*t*-Test	Cinn		*P*-values: not determined (neutral interaction)			*P* < 0.1
	AMB					*P* < 0.1

*MFCs are concentrations where ≥99.9% fungal death was achieved, except where noted in the table*.

*Cinn, cinnamaldehyde (mM); AMB, amphotericin B (μg mL^−1^); MIC, minimum inhibitory concentration; MFC, minimum fungicidal concentration; FICI, fractional inhibitory concentration indices; FFCI, fractional fungicidal concentration indices.

## Discussion

All compounds tested, except for 3,5-DMBA, are known natural volatiles or components of the essential oils of a number of plants, including almond and vanilla. Both 2,3- and 2,5-DHBAs and *trans-cinnamaldehyde* have been shown to have a moderate level (MICs 20–80 μg mL^−1^) of antibacterial activity (Wong et al., 2008). However, we found that the antifungal activity of these compounds, alone, is not particularly noteworthy.

However, as previously reported, certain phenolic antioxidants can prolong the activity of AMB against *C. albicans* by stabilizing the multiple double bonds of the polyene moiety. But, the mechanism by which the combination of such phenolics and AMB resulted in a synergistic interaction was unidentified (Beggs et al., 1978). Our results showed the DHBAs also augmented efficacy of AMB, *in vitro*, against yeast pathogens. Co-application of DHBAs with AMB resulted in complete inhibition of fungal growth at lower doses than any of the individual components applied, alone. Based on gene deletion mutant bioassays, it now appears that this synergy between AMB and DHBAs is by targeting at least one common cellular component in the antioxidant system, Mn-SOD. SODs of *C. albicans* are involved in biofilm persistence against miconazole (Bink et al., 2011), further demonstrating the role of fungal SODs in drug resistance. The non-DHBAs tested were poor chemosensitizing agents of AMB against yeast pathogens, indicating hydroxyl (−OH) substituents on the aromatic ring contributed to improved antifungal/chemosensitizing activity. Of note is benzaldehydes having *ortho-* and *para*-hydroxylation possessed higher antifungal activity than *meta-* or mono-hydroxyl analogs (Kim et al., 2008).

The results of this *in vitro* study demonstrate that chemically targeting the oxidative stress response system of fungi effectively augments antimycotic potency of AMB. DHBAs or their analogs could be developed as potent chemosensitizers to AMB in yeast pathogens. Chemosensitization by using natural compounds could enhance the efficacy of AMB to inhibit fungal growth, and lower the adverse side effects of AMB. Further *in vivo* studies are needed to determine if the activities of chemosensitizers shown in this *in vitro* study can translate to a clinically effective resolution of mycoses.

## Conflict of Interest Statement

The authors declare that the research was conducted in the absence of any commercial or financial relationships that could be construed as a potential conflict of interest.

## Abbreviations

AMB, amphotericin B; CFU, colony forming unit; Cinn, cinnamaldehyde; DHBA, dihydroxybenzaldehyde; 2,3-DHBA, 2,3-dihydroxybenzaldehyde; 2,5-DHBA, 2,5-dihydroxybenzaldehyde; 3,5-DMBA, 3,5-dimethoxybenzaldehyde; DMSO, dimethyl sulfoxide; EUCAST, European Committee on Antimicrobial Susceptibility Testing; FFCI, fractional fungicidal concentration indices; FICI, fractional inhibitory concentration indices; Glr1, glutathione reductase; Gsh1, γ-glutamylcysteine synthetase; LAMB, liposomal amphotericin B; MFC, minimum fungicidal concentration; MIC, minimum inhibitory concentration; Mn-SOD, mitochondrial superoxide dismutase; SG, synthetic glucose; Sod, superoxide dismutase; Sod1, cytosolic superoxide dismutase; Sod2, mitochondrial superoxide dismutase; Trx2, thioredoxin; WT, wild type; Ycf1, glutathione *S*-conjugate pump.
